# Proximity extension assay testing reveals novel diagnostic biomarkers of atypical parkinsonian syndromes

**DOI:** 10.1136/jnnp-2018-320151

**Published:** 2019-03-13

**Authors:** Edwin Jabbari, John Woodside, Tong Guo, Nadia K Magdalinou, Viorica Chelban, Dilan Athauda, Andrew J Lees, Thomas Foltynie, Henry Houlden, Alistair Church, Michele TM Hu, James B Rowe, Henrik Zetterberg, Huw R Morris

**Affiliations:** 1 Department of Clinical and Movement Neurosciences, UCL Queen Square Institute of Neurology, University College London, London, UK; 2 Reta Lila Weston Institute, UCL Queen Square Institute of Neurology, University College London, London, UK; 3 Queen Square Brain Bank for Neurological Disorders, UCL Queen Square Institute of Neurology, University College London, London, UK; 4 Department of Neurology, Royal Gwent Hospital, Newport, UK; 5 Nuffield Department of Clinical Neurosciences, University of Oxford, Oxford, UK; 6 Department of Clinical Neurosciences and MRC Cognition and Brain Sciences Unit, University of Cambridge, Cambridge, UK; 7 Department of Neurodegenerative Disease, UCL Queen Square Institute of Neurology, University College London, London, UK; 8 Institute of Neuroscience and Physiology, Department of Psychiatry and Neurochemistry, Sahlgrenska Academy, University of Gothenburg, Gothenburg, Sweden

**Keywords:** corticobasal degeneration, diagnostic test assessment, progressive supranuclear palsy, multiple system atrophy, cerebrospinal fluid

## Abstract

**Objective:**

The high degree of clinical overlap between atypical parkinsonian syndromes (APS) and Parkinson’s disease (PD) makes diagnosis challenging. We aimed to identify novel diagnostic protein biomarkers of APS using multiplex proximity extension assay (PEA) testing.

**Methods:**

Cerebrospinal fluid (CSF) samples from two independent cohorts, each consisting of APS and PD cases, and controls, were analysed for neurofilament light chain (NF-L) and Olink Neurology and Inflammation PEA biomarker panels. Whole-cohort comparisons of biomarker concentrations were made between APS (n=114), PD (n=37) and control (n=34) groups using logistic regression analyses that included gender, age and disease duration as covariates.

**Results:**

APS versus controls analyses revealed 11 CSF markers with significantly different levels in cases and controls (p<0.002). Four of these markers also reached significance (p<0.05) in APS versus PD analyses. Disease-specific analyses revealed lower group levels of FGF-5, FGF-19 and SPOCK1 in multiple system atrophy compared with progressive supranuclear palsy and corticobasal syndrome. Receiver operating characteristic curve analyses suggested that the diagnostic accuracy of NF-L was superior to the significant PEA biomarkers in distinguishing APS, PD and controls. The biological processes regulated by the significant proteins include cell differentiation and immune cell migration. Delta and notch-like epidermal growth factor-related receptor (DNER) had the strongest effect size in APS versus controls and APS versus PD analyses. DNER is highly expressed in substantia nigra and is an activator of the NOTCH1 pathway which has been implicated in the aetiology of other neurodegenerative disorders including Alzheimer’s disease.

**Conclusions:**

PEA testing has identified potential novel diagnostic biomarkers of APS.

## Introduction

The atypical parkinsonian syndromes (APS) include progressive supranuclear palsy (PSP), corticobasal syndrome (CBS) and multiple system atrophy (MSA). The high degree of clinical overlap between APS and Parkinson’s disease (PD) makes diagnosis challenging, and pathological diagnosis remains the gold standard.^1^ However, as we enter a new era of potential disease-modifying therapies,^2^ early and accurate diagnosis has never been more important.

Neurofilament light chain (NF-L), a marker of axonal degeneration of large-calibre myelinated axons, is a reliable differentiator of APS, PD and controls in cerebrospinal fluid (CSF)^3 4^ and plasma.^5^ However, the level of NF-L does not differentiate between PSP, CBS and MSA,^3^ and it is also raised in other neurodegenerative disorders such as frontotemporal dementia (FTD).^6^

Proximity extension assay (PEA) technology is a 96-plex immunoassay for high-throughput fluid protein biomarker detection, using unique antibody–oligonucleotide protein binding for quantitative real-time polymerase chain reaction (PCR)-based measurement.^7^ PEA biomarker measurement has previously been explored in neurological conditions such as traumatic brain injury.^8^ In addition, PEA appears to be less affected by the technical issues of multiplex enzyme-linked immunosorbent assay (ELISA) such as antibody cross-reactivity and inter-assay variability.^9^

We have used PEA biomarker measurement to identify diagnostic markers of APS that offer novel biological insights into these disorders. In particular, our study explores the role of inflammation which has been identified as a key component in the pathogenesis of Alzheimer’s disease (AD) and FTD via microglial activation pathways in biomarker^10^ and genetic^11^ studies.

## Methods

### Standard protocol approvals, registrations and patient consents

Two prospective cohorts, each consisting of subjects with PSP, CBS, MSA and PD, and controls, were recruited and followed up longitudinally. Cohort 1 was recruited at The National Hospital for Neurology and Neurosurgery, London, between 2012 and 2015 (Research Ethics Committee reference—12/LO/0640) for a Queen Square biomarker study of patients with APS.^3^ Cohort 2 was recruited between 2015 and 2017 as part of their involvement in the PROSPECT-UK study (Research Ethics Committee reference—14/LO/1575), a longitudinal observational study of patients with APS in the UK. Cohort 2 PD subject samples and clinical data were obtained from PD patients in the placebo arm of the Exenatide trial (trial approval by Brent NHS Research Ethics Committee, London).^12^ Patient consent for the trial covered the use of samples and clinical data in related studies such as this one. CSF sampling, quality control and storage protocols implemented in the trial were identical to the protocol outlined below.

Patients were assigned diagnoses according to current clinical diagnostic criteria. Of note, our application of the Movement Disorder Society (MDS) PSP diagnostic criteria^13^ identified probable PSP cases with clinical syndromes other than classical Richardson syndrome (RS), such as PSP-parkinsonism (PSP-P) and pure akinesia with gait freezing (PAGF). Baseline PSP rating scale (PSPRS) scores were obtained from patients with PSP and CBS on the same day of, but prior to, lumbar puncture (LP) testing. For each patient, the following clinical data were recorded: gender, age at motor symptom onset, date of motor symptom onset, age at the point of LP, disease duration at the point of LP, alive/deceased status of subjects at the point of censoring (7 December 2018) and the total disease duration in deceased subjects (defined as date of motor symptom onset to the date of death). In addition, a thorough review of current clinical notes for all patients was carried out to ensure that their clinical diagnosis had not changed. A subset of deceased patients underwent postmortem examination at the Queen Square Brain Bank, London, for neuropathological confirmation of diagnosis.

### LP/blood testing, sample handling and initial biomarker testing

Cases and controls underwent baseline LP testing. Cases and controls from cohort 1 also underwent venepuncture for blood testing at the same time as LP. CSF and blood samples were frozen and stored at −80ᵒC within 1 hour of sampling. Biomarker testing was performed on 0.5 mL aliquots and no aliquots had undergone interim freeze–thaw cycles. Blood-contaminated CSF samples (>500 red blood cells/μL) were excluded. Prior to PEA testing, CSF levels of the following markers were obtained using a separate aliquot of CSF: total-tau/phosphorylated-tau/Aβ1–42 (INNOTEST ELISA—Fujirebio Europe N.V., Gent, Belgium) and NF-L (Simoa platform; Quanterix, Lexington, Massachusetts, USA). Samples from cohorts 1 and 2 were analysed on separate runs for the above biomarkers.

### PEA testing

Biomarker panel testing was performed using multiplex PEA technology as previously described by Olink (Uppsala, Sweden).^7^ Samples were simultaneously run on two panels, (1) Neurology and (2) Inflammation, each consisting of 92 biomarkers, with 96 samples tested simultaneously on each run. Olink panel validation data are freely available online (https://www.olink.com/data-you-can-trust/validation/). The biological function of PEA markers of interest was obtained from the UniProt database (www.uniprot.org). Tissue expression of PEA markers of interest was assessed using publicly available data on the GTEx database (www.gtexportal.org). The GTEx database consists of 8555 samples from 53 tissues (including 13 brain regions) of 544 donors for which RNAseq was conducted. The GTEx Project was supported by the Common Fund of the Office of the Director of the National Institutes of Health, and by NCI, NHGRI, NHLBI, NIDA, NIMH and NINDS. The data used for the analyses described in this manuscript were obtained from the GTEx Portal on 31 July 2018.

Samples from cohorts 1 (CSF and plasma) and 2 (CSF alone) were analysed on separate runs. The resulting data for each biomarker were given as a normalised protein expression (NPX) value. NPX is an arbitrary unit on a Log2 scale with data being normalised to minimise both intra-assay and inter-assay variation. We subsequently performed intensity normalisation (detailed description at www.olink.com/content/uploads/2018/05/Data-normalization-and-standardization_v1.0.pdf) of CSF NPX values across cohorts 1 and 2 to allow the combination of data sets to carry out whole-cohort analyses.

### Statistical analyses

All of the following statistical analyses were performed using Stata V.14.0. Figures were generated using Stata V.14.0 and R V.3.3.2. Group comparisons of baseline clinical data (continuous variables) were carried out using t-tests, with statistical significance defined as p value <0.05. All protein biomarkers that had a >5% subject failure rate and all subjects that had a >5% marker failure rate were excluded from analyses.

Group comparisons of intensity-normalised PEA marker data were carried out using two separate approaches. First, we combined subjects with PSP and CBS to form a ‘tau’ group, and subjects with MSA and PD combined to form a ‘synuclein’ group in order to carry out whole-cohort tau versus synuclein group comparisons with gender, age at the point of testing and disease duration at the point of testing as covariates. Second, subjects with PSP, CBS and MSA were combined to form one ‘APS’ group in order to carry out whole-cohort APS versus controls and APS versus PD group comparisons. Initially, whole-cohort APS versus controls analyses for all PEA markers and NF-L were carried out using logistic regression analyses, with gender and age at the point of testing as covariates. We then carried out whole-cohort APS versus PD group analyses for NF-L and all PEA markers that had reached significance in the whole-cohort APS versus controls analyses. For APS versus PD comparisons, we used logistic regression analyses, with gender, age at the point of testing and disease duration at the point of testing as covariates. The threshold for significant group differences in both analyses was set using the Benjamini-Hochberg correction method for multiple testing^14^ with a false discovery rate of 5%. We calculated disease-specific association statistics for all PEA markers that reached significance in whole-cohort APS versus controls and APS versus PD analyses.

We carried out separate receiver operating characteristic (ROC) curve analyses for whole-cohort APS versus controls and APS versus PD group comparisons. In each analysis, ROC curves were generated for the following variables: (1) covariates; (2) CSF NF-L; (3) combined significant CSF PEA markers; (4) covariates+CSF NF-L+combined significant CSF PEA markers.

In subjects with APS, the relationship between the levels of significant PEA markers and (1) NF-L levels (log-transformed) and (2) PSPRS scores was assessed using linear regression analyses, with gender, age at the point of testing and disease duration at the point of testing as covariates.

## Results

A total of 151 cases and 34 controls were recruited to the study across two independent cohorts (table 1). All subjects underwent baseline CSF sampling for NF-L and PEA marker testing. All subjects from cohort 1 underwent paired blood sampling for plasma PEA marker testing. Of note, we excluded eight patients with CBS who fulfilled criteria for a CSF profile that was indicative of underlying AD pathology—defined as a CSF tau:Aβ1–42>1.^15^ In addition, all PSP cases from cohort 1 fulfilled probable RS criteria while the breakdown of cohort 2 PSP cases fulfilling probable criteria were as follows: 15 RS; 4 PSP-P; 2 PAGF.

**Table 1:** Summary of baseline clinical characteristics, CSF NF-L and clinical progression data

	Cohort 1					Cohort 2				
	PSP (n=33)	CBS (n=11)	MSA (n=29)	PD (n=25)	CON (n=30)	PSP (n=21)	CBS (n=11)	MSA (n=9)	PD (n=12)	CON (n=4)
Gender male/female (%)	48/52	57/43	55/45	64/36	50/50	81/19	18/82	89/11	67/33	25/75
Age at motor symptom onset (years)—mean, SD	65.1* 6.2	64.5* 8.0	60.2* 5.9	55.8‡ 8.1		63.6* 6.2	59.4* 7.8	61.0* 9.2	50.7‡ 6.0	
Age at LP (years)—mean, SD	69.6† 5.9	68.4† 8.4	64.4† 5.8	67.4†‡ 9.1	59.8* 9.9	67.2* 5.7	64.1* 7.5	65.6* 8.7	59.9†‡ 6.1	65.0* 2.9
Disease duration at LP (years)—mean, SD	4.5* 2.5	3.9* 1.2	4.2* 2.2	11.6 6.1		3.6* 2.0	4.7* 1.9	4.6* 2.6	9.2 3.1	
PSPRS score—mean, SD	42.0‡ 11.5	38.9 15.2				30.6‡ 9.1	30.4 10.8			
CSF NF-L concentration (ng/L)—mean, SD	2225.2 *†‡§ 913.4	2268.6 *† 1291.4	2991.0 *†‡ 1462.2	963.0 † 565.8	630.6 * 278.9	3228.5 *†‡ 1611.3	2547.3 *†§ 1255.7	4671.6 *†‡ 2768.9	980.3 218.7	868.5 236.4
% of subjects deceased at point of censoring	88	64	86	48		24	0	0	0	
Total disease duration in deceased group (years)—mean, SD	7.2* 2.5	7.0* 2.5	7.1* 2.4	13.7 6.8		6.0 2.3	NA	NA	NA	
No of pathologically confirmed cases	9 PSP=9	2 CBD=2	6 MSA=5 PSP=1	0		2 PSP=2	NA	NA	NA	

Only statistically significant (p<0.05) differences between continuous variables are noted from t-tests.

*vs PD in same cohort.

†vs controls in same cohort.

‡vs same measure in other cohort.

§vs MSA in same cohort.

CBS, corticobasal syndrome; CON, controls; CSF, cerebrospinal fluid; LP, lumbar puncture; MSA, multiple system atrophy; NA, data not available; NF-L, neurofilament light chain; PD, Parkinson’s disease; PSP, progressive supranuclear palsy; PSPRS, PSP rating scale.

CSF samples from all subjects underwent PEA marker testing for 184 markers (92 from the Neurology panel and 92 from the Inflammation panel). In total, 119/184 biomarkers (65 from the Neurology panel and 54 from the Inflammation panel) were detectable in the CSF of >95% of all subjects in both cohorts and were therefore used for further analyses. All subjects had a detectable result for >95% of the remaining 119 biomarkers. We carried out intensity normalisation across both cohorts to enable the combination of data sets to conduct whole-cohort analyses.

**Figure 1:**
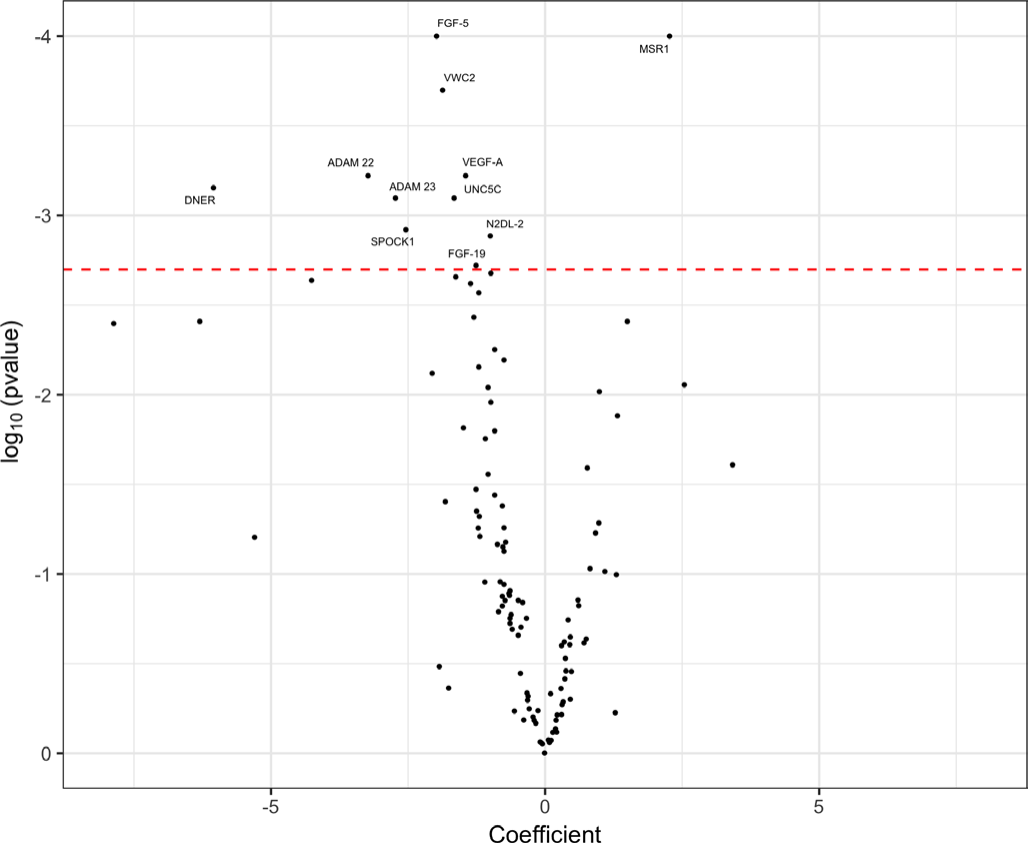
Volcano plot of cerebrospinal fluid proximity extension assay biomarkers, highlighting significant markers that differentiated the atypical parkinsonian syndromes (APS) group from controls. Markers to the right of 0 on the x-axis were higher in the APS group and markers to the left of 0 on the x-axis were higher in controls. The threshold for p value significance (<0.002) was set using the Benjamini-Hochberg correction method for multiple testing with a false discovery rate of 5%.

Our initial analyses did not reveal any markers that reached statistical significance in differentiating between tau and synuclein groups. We then combined PSP, CBS and MSA groups into one APS group. The ability of each biomarker to differentiate between the APS group and controls was assessed by carrying out individual logistic regression analyses that used gender and age at the point of testing as covariates. The resulting coefficient values, used as markers of group fold change, and p values were used to construct a volcano plot to highlight significant markers (figure 1). We identified 11 markers that reached statistical significance.

**Figure 2:**
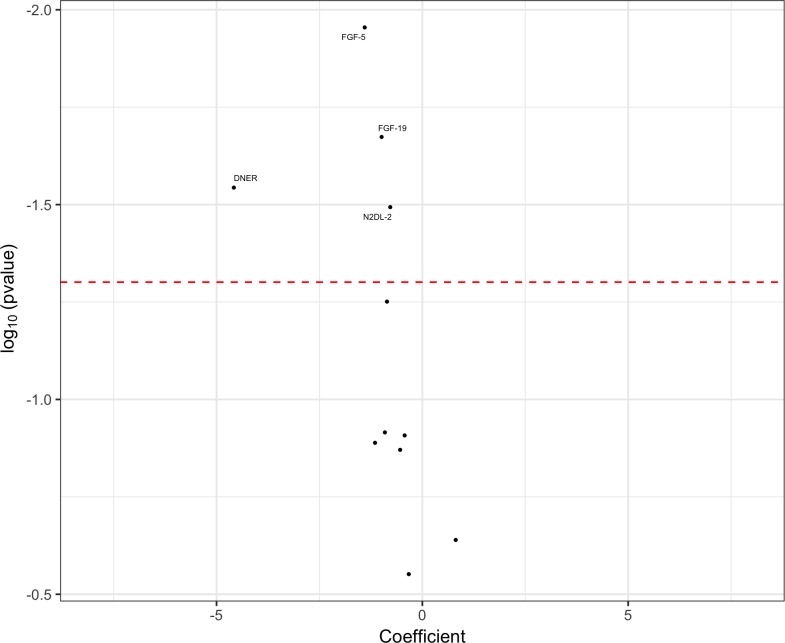
Volcano plot of cerebrospinal fluid proximity extension assay biomarkers, highlighting significant markers that differentiated the atypical parkinsonian syndromes (APS) group from the Parkinson’s disease (PD) group. Markers to the right of 0 on the x-axis were higher in the APS group and markers to the left of 0 on the x-axis were higher in the PD group. The threshold for p value significance (<0.05) was set using the Benjamini-Hochberg correction method for multiple testing with a false discovery rate of 5%.

We took forward the 11 significant markers from the whole-cohort APS versus controls analyses and assessed their ability to differentiate the APS group from the PD group by carrying out logistic regression analyses that used gender, age at the point of testing and disease duration at the point of testing as covariates (figure 2). We identified four markers with reduced CSF concentrations in APS cases as compared with PD cases, which were also reduced in APS versus controls. Although the remaining seven markers followed the same trends as in the APS versus controls analyses, these group differences did not reach statistical significance.

We did not detect any statistically significant (p<0.05) APS versus controls or APS versus PD differences using cohort 1 plasma data when carrying out logistic regression analyses using the same covariates as in the CSF analyses above.

We assessed for disease-specific differences between PSP, CBS, MSA, PD and control groups in the significant PEA markers by carrying out whole-cohort logistic regression analyses using gender, age at testing and disease duration at testing as covariates. Although we found significantly different levels of our identified markers between controls and each of the PSP, CBS and MSA groups, we found that the APS versus PD signals for FGF-5, FGF-19 and DNER were primarily being driven by lower levels in the MSA group. In addition, we found significantly lower group levels of FGF-5, FGF-19 and SPOCK1 in MSA compared with PSP and CBS (table 2).

**Table 2:** Disease-specific summary statistics of significant PEA CSF biomarkers

CSF biomarker	PSP (n=54) mean (NPX) SD	CBS (n=22) mean (NPX) SD	MSA (n=38) mean (NPX) SD	PD (n=37) mean (NPX) SD	CON (n=34) mean (NPX) SD
FGF-5	3.26*† 0.54	3.28*† 0.52	2.93*†§¶ 0.58	3.60† 0.53	3.52†§¶ 0.46
MSR1	2.11* 0.40	2.08*† 0.39	2.02* 0.48	1.95* 0.50	1.58†‡§¶ 0.48
VWC2	4.78* 0.49	4.93 0.55	4.55*‡ 0.54	5.13† 0.51	5.06†§ 0.51
VEGF-A	9.24* 0.46	9.34 0.54	8.98*‡ 0.53	9.61† 0.46	9.48†§ 0.55
ADAM22	7.64*‡ 0.29	7.67 0.24	7.56*‡ 0.26	7.84†§ 0.25	7.78†§ 0.26
DNER	10.02* 0.15	10.00* 0.17	9.96*‡ 0.16	10.11† 0.14	10.08†§¶ 0.13
UNC5C	2.14* 0.51	2.26 0.51	2.09*† 0.51	2.43† 0.43	2.46†§ 0.47
ADAM23	3.00* 0.30	3.04 0.34	2.89*‡ 0.33	3.21† 0.26	3.17†§ 0.28
SPOCK1	7.15*†‡ 0.29	7.12† 0.37	6.94*‡§¶ 0.32	7.36†§ 0.29	7.23†§ 0.29
N2DL-2	4.13* 0.72	3.92*‡ 0.71	3.90*‡ 0.85	4.60†¶ 0.74	4.52†§¶ 0.86
FGF-19	4.53*† 0.55	4.70† 0.53	4.23*‡§¶ 0.59	4.90† 0.51	4.79†§ 0.54

Only statistically significant (p<0.05) differences are noted from logistic regression analyses.

*vs controls.

†vs MSA.

‡vs PD.

§vs PSP.

¶vs CBS.

CBS, corticobasal syndrome; CON, controls; CSF, cerebrospinal fluid; MSA, multiple system atrophy; NPX, normalised protein expression value; PD, Parkinson’s disease; PEA, proximity extension assay; PSP, progressive supranuclear palsy.

The biological function and tissue expression of the significant PEA markers are summarised below (table 3).

**Table 3:** Biological function (UniProt database) and tissue expression (GTEx database) of significant PEA CSF biomarkers

CSF biomarker	Olink panel	Associated gene (chromosomal location)	Biological function	Tissue with highest expression (median TPM)	Brain region with highest expression (median TPM)
FGF-5 (fibroblast growth factor 5)	Inflammation	FGF5 (4q21.21)	Regulation of cell proliferation and cell differentiation	Fibroblasts (18.9)	Cerebellum (6.0)
MSR1 (macrophage scavenger receptor 1)	Neurology	MSR1 (8p22.1)	A membrane glycoprotein implicated in the pathological deposition of cholesterol in arterial walls during atherogenesis	Lung (33.2)	Hypothalamus (1.0)
VWC2 (von Willebrand factor C domain-containing protein 2)	Neurology	VWC2 (7p12.2)	Bone morphogenetic protein antagonist which may play a role in neural development	Cerebellum (24.4)	Cerebellum (24.4)
VEGF-A (vascular endothelial growth factor A)	Inflammation	VEGFA (6p21.1)	Growth factor active in angiogenesis, vasculogenesis and endothelial cell growth	Thyroid (613.1)	Cerebellum (39.7)
ADAM22 (disintegrin and metalloproteinase domain-containing protein 22)	Neurology	ADAM22 (7p21.12)	Regulation of cell adhesion and inhibition of cell proliferation	Cerebellum (87.4)	Cerebellum (87.4)
DNER (delta and notch-like epidermal growth factor-related receptor)	Inflammation	DNER (2q36.3)	Activator of the NOTCH1 pathway. May mediate neuron–glia interaction during astrocytogenesis	Substantia nigra (124.9)	Substantia nigra (124.9)
UNC5C (netrin receptor)	Neurology	UNC5C (4q22.3)	Mediates axon repulsion of neuronal growth cones in the developing nervous system	Thyroid (11.7)	Cervical cord (8.2)
ADAM23 (disintegrin and metalloproteinase domain-containing protein 23)	Neurology	ADAM23 (2q33.3)	May play a role in cell–cell and cell–matrix interactions	Frontal cortex (45.5)	Frontal cortex (45.5)
SPOCK1 (testican 1)	Neurology	SPOCK1 (5q31.2)	May play a role in cell–cell and cell–matrix interactions	Cerebellum (314.3)	Cerebellum (314.3)
N2DL-2 (UL16-binding protein 2)	Neurology	ULBP2 (6q25.1)	Binds and activates the KLRK1/NKG2D receptor, mediating natural killer cytotoxicity	Fibroblasts (9.6)	Cerebellum (5.0)
FGF-19 (fibroblast growth factor 19)	Inflammation	FGF19 (11q13.3)	Involved in the suppression of bile acid biosynthesis through downregulation of CYP7A1 expression	Testis (0.5)	Cerebellum (0.4)

CSF, cerebrospinal fluid; PEA, proximity extension assay; TPM, transcripts per kilobase million.

**Figure 3:**
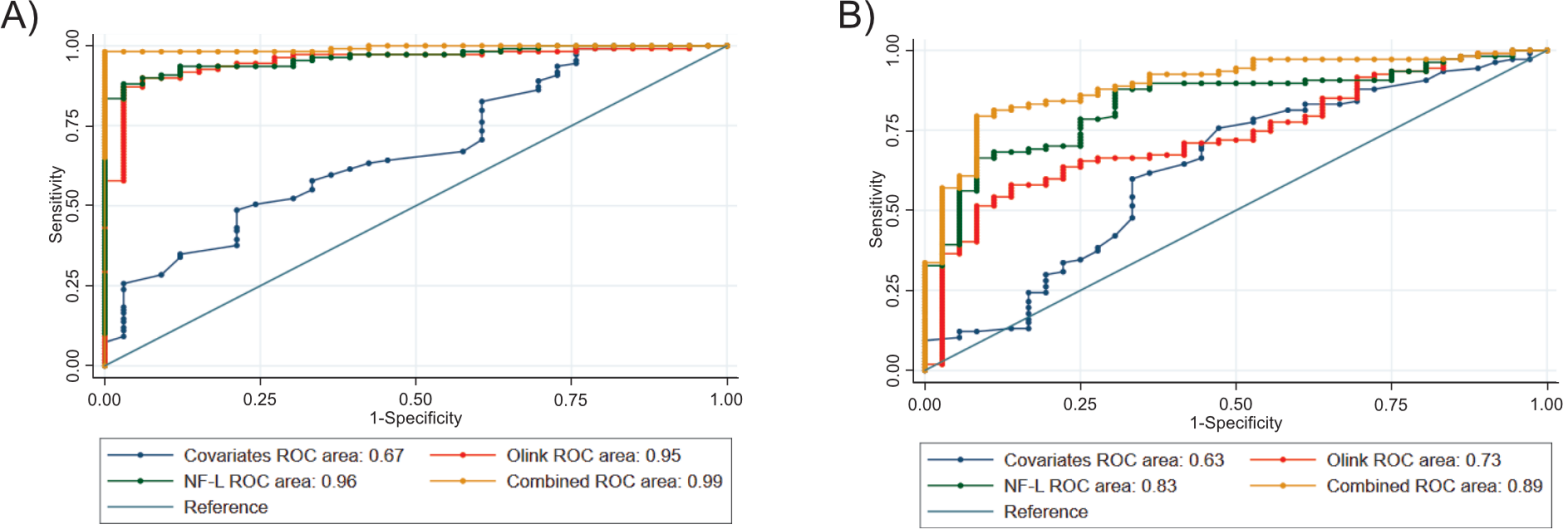
Receiver operating characteristic (ROC) curve analyses of: (A) atypical parkinsonian syndromes (APS) vs controls; (B) APS vs Parkinson’s disease (PD). Combined plot=significant Olink proximity extension assay markers+neurofilament light chain (NF-L)+covariates. Covariates in APS vs controls analysis=age at testing and gender. Covariates in APS vs PD analysis=age at testing, disease duration at testing and gender.

We carried out separate whole-cohort APS versus controls and APS versus PD ROC curve analyses to assess the diagnostic strength of the significant PEA markers in comparison with NF-L (figure 3).

Using whole-cohort APS group data, we did not detect any statistically significant relationships between the CSF levels of the significant PEA markers and both log-transformed CSF NF-L levels and PSP rating scale scores by conducting linear regression analyses using gender, age at testing and disease duration at testing as covariates.

## Discussion

Our study highlights the application of high-throughput multiplex PEA testing to reveal novel biological insights into atypical parkinsonian disorders. The statistical methods used in the APS versus controls and APS versus PD analyses provides robust evidence for the diagnostic markers that we have identified.

The baseline clinical characteristics, CSF NF-L data and pathological diagnosis data suggest a high level of diagnostic accuracy in our two cohorts which enabled a combined whole-cohort study. Our thorough current clinical notes review for each case ensured that patients had not subsequently developed clinical features suggestive of alternative diagnoses. In particular, none of the cohort 1 PD group cases had evidence of oculomotor dysfunction suggesting inadvertent inclusion of PSP-P cases. The mean disease duration at the point of diagnosis in this group was 11.6 years, and at this stage we would certainly expect to see oculomotor dysfunction in patients with PSP-P.^16^ Similarly, patients with PSP-P included in the cohort 2 PSP group fulfilled ‘probable’ MDS PSP diagnostic criteria.^13^ This means that all cases would have had the presence of slowed vertical saccades and/or a vertical supranuclear gaze palsy, both of which are highly suggestive of underlying PSP pathology, such that we do not believe this group inadvertently contained PD cases.

In addition, we intensity-normalised our PEA biomarker data across both cohorts to reduce inter-run variability, allowing us to combine data sets for better powered whole-cohort analyses.

Although our initial approach of creating tau and synuclein groups led to more pathologically homogeneous group comparisons, this did not yield any significant results. However, our subsequent whole-cohort analyses identified 11 CSF biomarkers that differentiated APS from controls. Four of these markers (FGF-5, FGF-19, DNER and N2DL-2) also differentiated APS from PD, with levels of the remaining seven markers not reaching statistical significance but following the same trend as in the APS versus controls analyses. Despite having heterogeneous pathology within the combined APS group, it is possible that the markers reaching significance in the above analyses are, like NF-L, non-specific markers of more rapid rates of neurodegeneration seen in PSP, CBS and MSA in comparison with PD. However, it is more likely that we were underpowered to detect pathology-specific differences in biomarker concentrations when carrying out tau (PSP and CBS) versus synuclein (MSA and PD) analyses. This is suggested by the fact that we found significantly lower group levels of FGF-5, FGF-19 and SPOCK1 in MSA compared with PSP and CBS groups. It remains premature to suggest that these are disease-specific markers until further replication data in larger cohorts is obtained.

All but one of the significant PEA markers had lower levels in the APS group compared with both PD and controls. Although this trend draws parallels with the observation of lower CSF levels of Aβ1–42 in patients with AD compared with controls, thought to reflect the incorporation of Aβ1–42 into amyloid plaques,^17^ it is unclear as to why this is the case in our identified markers.

The biological processes regulated by the significant PEA markers include cell proliferation/differentiation, cell apoptosis, immune cell migration and neural development. Of particular interest, DNER had the strongest effect size (coefficient) of all of the markers in both APS versus controls and APS versus PD analyses. DNER is highly expressed in substantia nigra, is an activator of the NOTCH1 pathway which has a role in neuronal and glial cell differentiation, and has previously been implicated in the aetiology of AD.^18^

APS versus controls and APS versus PD ROC curve analyses revealed that the individual area under the curve values for CSF NF-L alone were superior to the combination of the significant PEA markers.

There is strong evidence that levels of NF-L can track and predict the rate of disease progression in PSP.^19^ The lack of a linear relationship between the levels of the significant PEA markers and both the level of NF-L and PSPRS scores suggest that these markers are unlikely to have prognostic value. However, this needs to be explored further with longitudinal biomarker measurements to assess the temporal pattern of these markers in relation to changing clinical rating scale scores.

In summary, we present promising findings using PEA biomarker technology to discover novel diagnostic markers of APS. Although outside of the scope of this study, follow-up work includes replication of our findings in larger cohorts of subjects with APS which, in turn, may lead to the discovery of disease-specific and pathology-specific markers. A similar approach using phenotype group comparisons such as RS versus PSP-P/PAGF would also be of interest. In addition, we would aim to validate PEA as a reliable multiplex technique by comparing the levels of markers measured by PEA, single-molecule array and ELISA.
